# The Human EKC/KEOPS Complex Is Recruited to Cullin2 Ubiquitin Ligases by the Human Tumour Antigen PRAME

**DOI:** 10.1371/journal.pone.0042822

**Published:** 2012-08-13

**Authors:** Adalberto Costessi, Nawel Mahrour, Vikram Sharma, Rieka Stunnenberg, Marieke A. Stoel, Esther Tijchon, Joan W. Conaway, Ronald C. Conaway, Hendrik G. Stunnenberg

**Affiliations:** 1 Department of Molecular Biology, Nijmegen Centre for Molecular Life Sciences, Radboud University, Nijmegen, The Netherlands; 2 Stowers Institute for Medical Research, Kansas City, Missouri, United States of America; 3 Department of Biochemistry and Molecular Biology, Kansas University Medical Center, Kansas City, Kansas, United States of America; Institute of Genetics and Molecular and Cellular Biology, France

## Abstract

The human tumour antigen PRAME (preferentially expressed antigen in melanoma) is frequently overexpressed during oncogenesis, and high *PRAME* levels are associated with poor clinical outcome in a variety of cancers. However, the molecular pathways in which PRAME is implicated are not well understood. We recently characterized PRAME as a BC-box subunit of a Cullin2-based E3 ubiquitin ligase. In this study, we mined the PRAME interactome to a deeper level and identified specific interactions with OSGEP and LAGE3, which are human orthologues of the ancient EKC/KEOPS complex. By characterizing biochemically the human EKC complex and its interactions with PRAME, we show that PRAME recruits a Cul2 ubiquitin ligase to EKC. Moreover, EKC subunits associate with PRAME target sites on chromatin. Our data reveal a novel link between the oncoprotein PRAME and the conserved EKC complex and support a role for both complexes in the same pathways.

## Introduction

The human oncoprotein PRAME (preferentially expressed antigen in melanoma) was first identified and cloned as the antigen responsible for an anti-tumour immune response in a melanoma patient [1]. Follow-up experiments revealed that *PRAME* is expressed at low levels in few normal adult tissues like adrenals, ovaries, and endometrium, and at high levels only in the testis [1], [2]. However, overexpression of *PRAME* is frequently found in a wide variety of human cancers, including acute and chronic haematological tumours, synovial sarcoma, lung, breast, and renal carcinoma [1], [3]. Importantly, high *PRAME* levels were found to correlate with advanced stages of disease in melanoma [4], neuroblastoma [5], serous ovarian adenocarcinoma [6], and chronic myeloid leukaemia [7], and to constitute an independent prognostic factor of poor clinical outcome in breast cancer [8], [9]. In contrast, high levels of *PRAME* were found to correlate with good prognosis in leukaemia cases carrying the t(15;17) PML-RAR translocation (acute promyelocitic leukaemia) [10].

Although these findings suggested a role for PRAME in human malignancies, the detailed molecular mechanisms and pathways involved are not yet clear. PRAME was reported to repress retinoic acid signaling in melanoma cell lines [11], but this was not confirmed for breast cancer or leukaemia cases [9], [12]. Conflicting reports on leukaemia cells suggested that PRAME might induce caspase-independent cell death [13], or repress apoptosis-related genes to promote cell survival [14].

Recently, through biochemical characterization of PRAME-containing protein complexes, we established that this oncoprotein is a component of Cullin2-based E3 ubiquitin ligases and belongs to the family of BC-box proteins, associating PRAME to a clear biochemical activity and pathway [15]. PRAME establishes direct interactions with other ligase subunits through conserved N-terminal motifs: a BC-box (aa. 25–34) mediates interactions with the ElonginB-ElonginC heterodimer, and a downstream Cul2-box (aa. 48–56) mediates interactions with the Cullin2 scaffold protein. Genome-wide chromatin immunoprecipitation experiments further revealed that Cul2-PRAME ubiquitin ligases specifically associate with active promoters regulated by the transcription factor NFY and with proximal enhancers [15].

Two independent laboratories have identified an ancient and highly conserved multiprotein complex named KEOPS [16] or EKC [17], which has orthologues from Archaea to Eukarya and has been implicated in telomeres maintenance, transcriptional regulation, and t^6^A modification of tRNAs. Yeast EKC comprises four subunits which are also conserved in the human genome (human orthologues are indicated in brackets): Pcc1p (LAGE3, also known as ESO3), the ATPase Kae1p (OSGEP), the kinase Bud32p (TP53RK, also known as PRPK), and Cgi121p (TPRKB). In addition, yeast EKC also includes Gon7p (also known as Pcc2p), which appears to be fungi-specific [17].

Intriguingly, the OSGEP subunit is also present in bacteria (YgjD) and eukaryotic genomes express an OSGEP paralogue (Qri7/OSGEPL1) that localizes to mitochondria [18]. Comparative genomic studies identified OSGEP as one of the very few genes present in all genomes sequenced so far [19], suggesting an extremely conserved function. Very recently, several research groups have reported a crucial role for the YgjD/Kae1/OSGEP protein family in the biosynthesis of N6-threonylcarbamoyl adenosine (t^6^A) [20]–[22]: a universal modification at position 37 of tRNAs decoding ANN codons, which is required for accurate translation of messenger RNAs [23].

Human LAGE3 belongs to the NY-ESO gene family together with the closely related LAGE1 and LAGE2 [24], and all three genes are clustered in the same region on chromosome X. While LAGE3 is ubiquitously expressed, LAGE1 and LAGE2 are cancer-testis antigens with high expression in healthy testis and upregulation in a number of cancer tissues, similarly to PRAME.

The aim of the present study was to mine the protein-protein interactome of the PRAME oncoprotein to a deeper level by protein complex purification and mass spectrometry. Our experiments revealed that PRAME specifically interacts with the EKC complex in human cells and that it recruits Cul2-based E3 ubiquitin ligases to EKC. We further show that EKC subunits co-localize with PRAME at the promoter regions of transcriptionally active human genes, supporting a common functional role for these complexes on chromatin. Overall, our findings provide novel and important insights in the molecular pathways in which the oncoprotein PRAME is active.

## Results

### PRAME Interacts with the EKC/KEOPS Complex

We have recently applied biochemical purification strategies and characterized PRAME as a component of Cullin2 E3 ubiquitin ligases [15]. Here, we improved the immunoaffinity purification protocols and mined the PRAME interactome to a deeper level (see materials and methods). We isolated epitope-tagged PRAME (StrepII-Myc-3xHA-PRAME, referred to as TAG-PRAME) from K562 cells, which express high levels of endogenous PRAME, and from HeLaS3 cells, which express endogenous PRAME at very low to undetectable levels. As expected, mass spectrometry analysis of HA immunoprecipitates detected Cullin2 ligase components with the highest emPAI: Cullin2, Elongin B, Elongin C, and Rbx1 (Table 1). Notably, western blot analysis showed that Cullin2 and Elongin C were expressed to similar levels in nuclear extracts from the two cell lines (not shown).

**Table 1:** PRAME-interacting proteins identified by Mass Spectrometry of purified complexes from K562 and HeLaS3 cells.

		K562 cells							HeLaS3 cells						
ProteinID	Proteindescription	AVGemPAI	PRAMEMYC 1	PRAMEMYC 2	PRAMEHA 1	PRAMEHA 2	PRAMEHA 3	CtrlMYC 1	CtrlMYC 2	Ctrl HA 1	CtrlHA 2	PRAMEnucl	PRAMEcyto	Ctrlnucl	Ctrlcyto
IPI00026670.3	**TCEB2 Transcription elongation factor B polypeptide 2**	7.00	5	4	8	7	6	0	2	1	0	6	8	2	2
IPI00300341.5	**TCEB1 Transcription elongation factor B polypeptide 1**	5.01	6	6	4	8	6	1	0	1	0	7	7	4	1
IPI00019282.1	**PRAME Melanoma antigen preferentially expressed in tumors**	4.01	6	12	27	18	12	0	0	0	0	18	30	0	0
IPI00014311.4	**CUL2 Cullin-2**	2.05	12	22	34	31	23	0	6	2	0	18	30	0	0
IPI00015809.1	**OSGEP Probable O-sialoglycoprotein endopeptidase**	0.91	4	3	6	7	6	0	0	0	1	1	6	0	0
IPI00032314.2	**LAGE3 L antigen family member 3**	0.89	1	0	3	3	3	0	0	0	0	1	4	0	0
IPI00000643.1	BAG2 BAG family molecular chaperone regulator 2	0.77	0	0	9	5	1	0	0	1	0	5	6	4	0
IPI00003386.3	**RBX1 RING-box protein 1**	0.62	1	0	1	1	1	0	1	0	0	0	0	0	0
IPI00395674.1	SNRPB Isoform SM-B of Small nuclear ribonucleoprotein-associated proteins B and B’	0.59	4	5	1	1	2	0	4	0	0	1	0	0	0
IPI00221089.5	RPS13 40S ribosomal protein S13	0.58	0	3	3	1	4	0	4	1	0	3	4	7	0
IPI00005658.3	UBL4A Ubiquitin-like protein 4A	0.57	0	0	5	2	3	0	0	2	0	7	4	2	0
IPI00020042.2	PSMC4 Isoform 1 of 26S protease regulatory subunit 6B	0.54	0	0	13	1	1	0	0	13	1	2	0	0	0
IPI00383296.5	HNRPM Isoform 2 of Heterogeneous nuclear ribonucleoprotein M	0.48	0	16	11	11	6	0	12	1	0	1	0	0	0
IPI00003881.5	HNRPF Heterogeneous nuclear ribonucleoprotein F	0.48	2	7	6	1	1	0	7	0	0	6	0	0	1
IPI00219153.4	RPL22 60S ribosomal protein L22	0.47	1	1	1	1	1	0	1	0	0	0	1	1	0
IPI00017381.1	RFC4 Replication factor C subunit 4	0.46	0	4	9	3	6	0	0	7	1	11	1	0	3
IPI00477313.3	HNRNPC Isoform C2 of Heterogeneous nuclear ribonucleoproteins C1/C2	0.45	2	6	5	2	3	0	1	3	0	0	1	2	0
IPI00456631.5	AOF2 Isoform 1 of Lysine-specific histone demethylase 1	0.38	0	4	14	12	1	0	0	1	0	6	0	3	0
IPI00554723.5	RPL10 60S ribosomal protein L10	0.34	0	0	4	2	2	0	0	3	0	2	11	4	0
IPI00013881.6	HNRPH1 Heterogeneous nuclear ribonucleoprotein H	0.32	3	5	4	3	1	1	3	0	0	6	0	3	1

PRAME-associating proteins were purified from nuclear extracts of K562-TAG-PRAME by anti-MYC or anti-HA immunoprecipitation and from HeLaS3-TAG-PRAME cells by anti-HA immunoprecipitation and subjected to mass spectrometry. Immunoprecipitations from wild type K562 and HeLaS cells were used as a control for immunospecificity of the purifications. Components of Cullin2 ligases and EKC complex are indicated in bold.

Furthermore, a number of additional proteins were specifically detected in eluates of K562-TAG-PRAME and HeLaS3-TAG-PRAME cells, but not in the control parental cell lines. These included OSGEP (O-sialoglycoprotein endopeptidase) and LAGE3 (L antigen family member 3), which are the human orthologues of the Kae1 and Pcc1 subunits of the recently described EKC complex (Table 1). OSGEP and LAGE3 were present with a similar abundance in TAG-PRAME eluates from both K562 and HeLaS3 cell lines, consistent with similar expression levels. However, in these eluates we did not identify peptides matching TP53RK or TPRKB, which are the predicted human orthologues of the EKC subunits Bud32 and Cgi121, respectively.

Other potential interactors identified in PRAME eluates were BAG2 and UBL4A (Table 1). BAG2 (BAG family molecular chaperone regulator 2) is a nucleotide exchange factor for the chaperone protein Hsp70 that contributes to the processing of misfolded proteins [25]. Similarly, UBL4A (Ubiquitin-like protein 4A) is part of a chaperone protein complex that promotes correct membrane targeting of tail-anchored proteins [26], [27]. Peptides for both BAG2 and UBL4A were identified in all anti-HA eluates from K562-TAG-PRAME cells, but were not detected in the anti-MYC eluates. Additional experiments are therefore needed to establish whether these proteins are real interactors of PRAME or rather false positives. For the purpose of this study, we further concentrated on EKC subunits.

**Figure 1:**
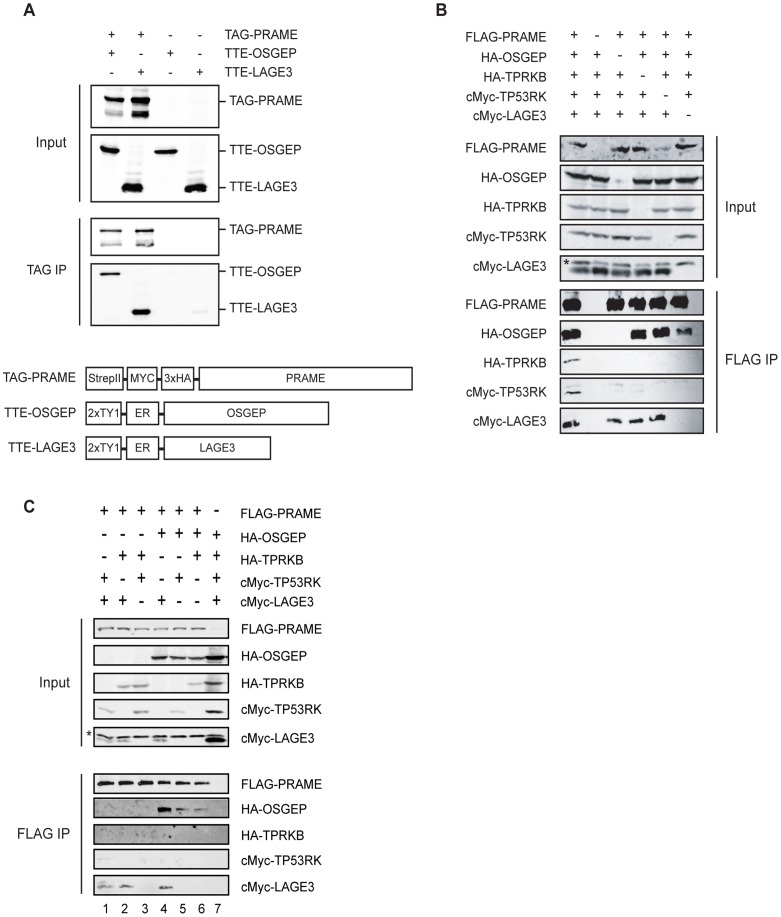
PRAME interacts with OSGEP and LAGE3. (A) Coimmunoprecipitation assays verify the interaction of PRAME with the EKC subunits OSGEP and LAGE3. Constructs expressing the indicated proteins were transiently transfected in 293T cells. Anti-TAG immunoprecipitations were performed with rabbit HA antibody (Abcam). TAG-PRAME was detected with monoclonal anti-HA (Covance) and TTE-tagged proteins with monoclonal BB2 (Diagenode) which recognizes the TY1 tag. The lower panel shows a scheme of the tagged proteins used (tags and coding sequences are not on scale). (B-C) PRAME directly interacts with EKC complex through OSGEP and LAGE3. SF21 cells were co-infected with baculoviruses expressing the indicated proteins. Anti-FLAG immunoprecipitations were performed, and total cell lysates and eluates were analyzed by western blotting. Panels showing anti-HA immunoblots are from the same exposure of the same blot, as are the panels showing anti-cMyc immunoblots. Asterisks indicate light chains of the antibodies used in the immunoprecipitation.

The specificity of the interactions between PRAME and EKC subunits was confirmed by transient transfections in HEK293T cells and coimmunoprecipitation experiments of TAG-PRAME with OSGEP and LAGE3 fused to a TTE (TY1-TY1-ER) epitope tag (Fig. 1A). We also used a baculovirus expression system to co-express FLAG-PRAME with different combinations of the four human EKC subunits in Sf21 insect cells. Consistent with the transient transfection experiments, we observed that PRAME could bind to OSGEP and LAGE3 independent of TP53RK and TPRKB. Interestingly, although both OSGEP and LAGE3 could bind PRAME independent of the other (Fig. 1B, lanes 3 and 6), LAGE3 appears to enhance the PRAME-OSGEP interaction, since substantially more OSGEP bound to PRAME when LAGE3 was also expressed (Fig 1B, compare lanes 1, 4, and 5 to lane 6, and Fig. 1C, compare lane 4 to lanes 5 and 6). Finally, we observed small amounts of TP53RK and TPRKB copurifying with FLAG-PRAME dependent on both OSGEP and LAGE3 (Fig. 1B, lanes 1, 3, and 6).

Taken together, our results identified stable interactions between PRAME and human orthologues of the EKC/KEOPS complex.

### EKC Purifications from K562 Cells

We next studied the expression patterns of EKC, Cullin2 ligase subunits and *PRAME* in normal and cancer cells by querying the GeneSapiens gene expression database [2]. This database contains mRNA expression data of most human genes across almost 10000 array experiments from 175 healthy and pathological tissues. Consistent with the literature, we found that *PRAME* is expressed only in very few normal adult tissues while it is upregulated in several cancers (Fig. S1). On the contrary, *LAGE3*, *OSGEP*, *TP53RK*, *TPRKB*, and *CUL2* seem to be expressed in most if not all tissues (Figures S1, S2, S3, S4).

To explore further the protein-protein interactions between PRAME and EKC, we generated K562 cell lines stably expressing epitope-tagged versions of each of the four human predicted human EKC subunits and performed protein complex purifications. As shown in Table 2, each EKC subunit co-purified with the other three subunits of the complex, indicating that a complete EKC complex is present in human cells.

**Table 2:** Human EKC-interacting proteins identified by Mass Spectrometry of purified complexes from nuclear extracts of K562 and HeLaS3 cells.

ProteinID	Proteindescription	K562LAGE3	K562OSGEP	K562TP53RK	K562TPRKB	HeLaLAGE3	K562ctrl 1	K562ctrl 2	K562ctrl 3	HeLactrl	K562LAGE3emPAI	K562OSGEPemPAI	K562TP53RKemPAI	K562TPRKBemPAI	HeLaLAGE3emPAI
IPI00032314.2	**LAGE3 L antigen family member 3**	6	8	8	6	7	0	0	0	0	4.62	9.00	9.00	4.62	6.50
IPI00015809.1	**OSGEP Probable O-sialoglycoprotein endopeptidase**	12	17	17	12	21	0	1	0	0	3.28	6.85	6.85	3.28	11.74
IPI00290305.3	**TP53RK TP53-regulating kinase**	8	10	15	9	7	0	0	0	0	2.73	4.18	10.79	3.39	2.16
IPI00301432.3	**TPRKB Isoform 1 of TP53RK-binding protein**	5	11	15	8	6	0	0	0	0	2.16	11.59	30.62	5.31	2.98
IPI00026670.3	**TCEB2 Transcription elongation factor B polypeptide 2**	3	8	1	2	2	1	0	4	3	1.68	12.90	0.39	0.93	0.93
IPI00014311.4	**CUL2 Cullin-2**	21	0	1	2	0	2	0	8	0	1.49	0.00	0.04	0.09	0.00
IPI00019282.1	**PRAME Melanoma antigen preferentially expressed in tumors**	6	18	3	0	0	0	0	0	0	0.74	4.25	0.32	0.00	0.00
IPI00646167.2	C14orf142 hypothetical protein LOC84520	1	2	1	0	3	0	0	0	0	0.59	1.51	0.59	0.00	2.98
IPI00007797.3	FABP5;FABP5L7 Fatty acid-binding protein, epidermal	2	0	0	1	0	0	0	2	0	0.59	0.00	0.00	0.26	0.00
IPI00005658.3	UBL4A Ubiquitin-like protein 4A	2	8	6	3	5	2	0	2	2	0.47	3.64	2.16	0.78	1.61
IPI00019912.3	HSD17B4 Peroxisomal multifunctional enzyme type 2	7	0	10	3	0	2	0	0	0	0.44	0.00	0.69	0.17	0.00
															
IPI00304903.4	SPRR1B Cornifin-B	1	0	0	0	0	0	0	0	0	0.39	0.00	0.00	0.00	0.00
IPI00742943.1	NUP43 Nucleoporin Nup43	2	0	0	0	1	0	0	0	0	0.36	0.00	0.00	0.00	0.17
IPI00300341.5	**TCEB1 Transcription elongation factor B polypeptide 1**	1	6	3	1	0	1	0	2	1	0.33	4.62	1.37	0.33	0.00
IPI00293434.2	SRP14 Signal recognition particle 14 kDa protein	1	2	3	0	1	0	0	2	0	0.33	0.78	1.37	0.00	0.33
IPI00446354.1	- CDNA FLJ41805 fis, clone NOVAR2000962	1	0	0	0	0	0	0	0	0	0.33	0.00	0.00	0.00	0.00
IPI00020025.3	SH3PXD2B CDNA FLJ20831 fis, clone ADKA03080	1	0	0	0	0	0	0	0	0	0.23	0.00	0.00	0.00	0.00
IPI00027626.3	CCT6A T-complex protein 1 subunit zeta	3	24	11	8	8	0	1	0	3	0.22	3.85	1.06	0.69	0.69
															
IPI00386765.2	PDE4D Isoform 7 of cAMP-specific 3′,5′-cyclic phosphodiesterase 4D	1	0	0	1	0	0	0	1	0	0.21	0.00	0.00	0.21	0.00
IPI00007423.1	ANP32B Isoform 1 of Acidic leucine-rich nuclear phosphoprotein 32 family member B	1	0	6	2	0	0	0	4	0	0.19	0.00	1.89	0.43	0.00

Proteins interacting with each of human EKC subunits were identified by anti-HA immunoprecipitations and mass spectrometry on the cell lines indicated. Components of Cullin2 ligases and EKC complex are indicated in bold.

Furthermore, TAG-OSGEP and TAG-LAGE3 (but not TAG-TP53RK or TAG-TPRKB) copurified with significant amounts of endogenous PRAME, consistent with TAG-PRAME purifications. Remarkably, TAG-OSGEP and TAG-LAGE3 associated also with Cullin2 ligase components (Table 2). The few peptides for Cul2-PRAME components identified in TAG-TP53RK and TAG-TPRKB eluates were not supported by western blot of the same samples (not shown).

Interestingly, peptides matching the putative protein C14orf142 were specifically identified in eluates from OSGEP, LAGE3 and TP53RK (Table 2). Similarly to LAGE3 and TPRKB, C14orf142 is predicted to be a small protein without clear structural domains. It is tempting to speculate that this protein might constitute a novel EKC interactor in human cells.

**Figure 2:**
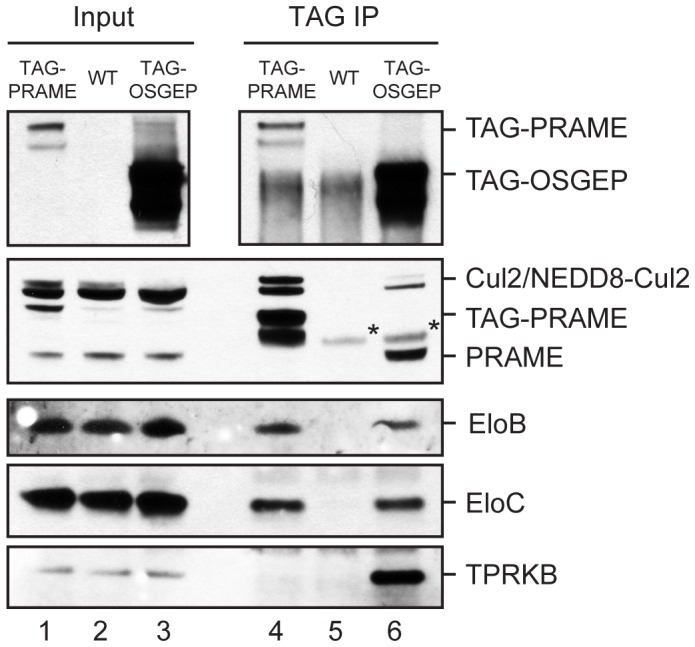
OSGEP interacts with PRAME and Cul2 ubiquitin complex components. OSGEP interacts with PRAME and Cul2-EloBC ligases. Immunoblot analysis of TAG-PRAME and TAG-OSGEP protein complexes purified from K562 cells to verify the mass spectrometry data. Mock purification was performed on wild type cells. 0.8% of input and 33% of IP were separated on NuPage 4–12% gels. Tagged proteins were detected with mouse HA antibody (Covance, top panel); endogenous PRAME and TAG-PRAME were detected in the second panel with affinity-purified PRAME antibody after staining for Cul2; the other proteins were detected as indicated. Asterisks indicate protein A that dissociated from the beads after elution.

Although we did not identify peptides matching Cul2 by mass spectrometry of TAG-OSGEP eluates, we did detect small amounts of Cul2 by western blotting of the same samples (Fig. 2, lane 6). Intriguingly, the level of Cul2 detected in TAG-OSGEP eluates was substantially lower than in TAG-PRAME eluates (Fig. 2, lanes 4 and 6), while Elongins B and C were present in similar amounts in all purifications. These findings might indicate that OSGEP establishes less stable interactions with Cullin2 as compared to PRAME. Consistent with the mass spectrometry results, TAG-PRAME did not copurify with TPRKB (Fig. 2, lane 4), while a clear band was detected in TAG-OSGEP eluates.

Our proteomic experiments show for the first time that a complete EKC complex is present in human cells, and that the OSGEP and LAGE3 subunits are present in a complex together with PRAME and Cullin2 ligase components.

### Topology of the human EKC complex

**Figure 3:**
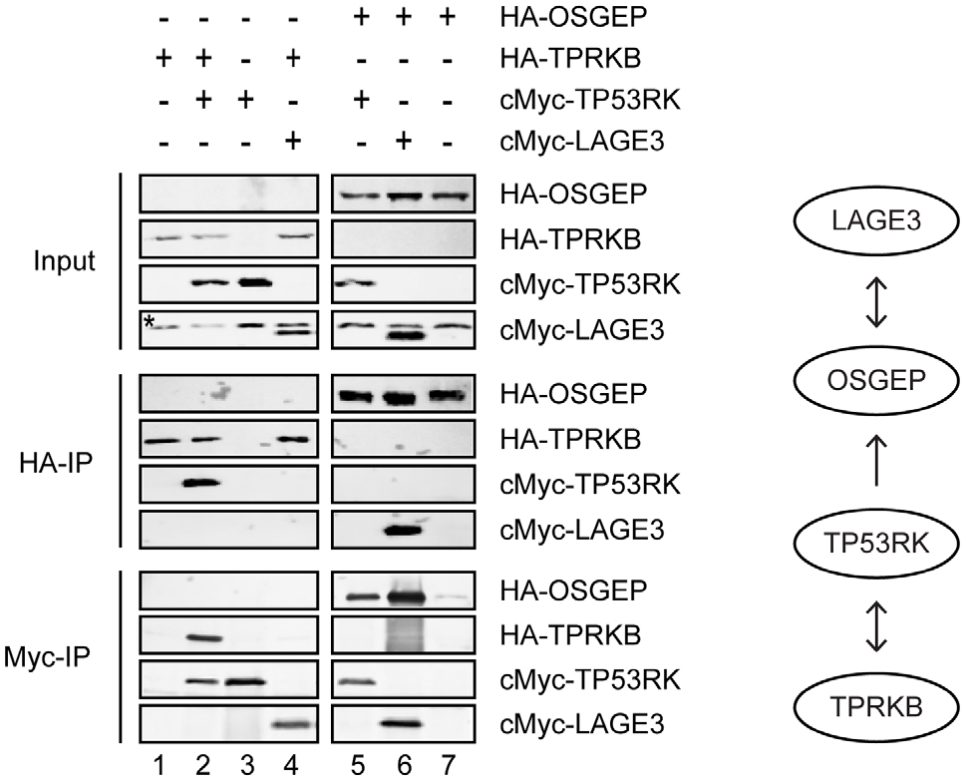
Organization of human EKC complex. SF21 cells were co-infected with baculoviruses expressing the indicated proteins. Anti-FLAG immunoprecipitations were performed, and total cell lysates and eluates were analyzed by western blotting. Interactions detected are summarized in the diagram on the right. Panels showing anti-HA immunoblots are from the same exposure of the same blot, as are the panels showing anti-cMyc immunoblots. Asterisk indicates light chains of the antibodies used in the immunoprecipitation.

In order to study the topology of the interactions within the human EKC complex, we expressed pairs of EKC subunits in the baculovirus system and performed immunoprecipitations (Fig. 3). As shown in figure 3, we observed reciprocal coimmunoprecipitation of HA-LAGE3 and c-Myc-OSGEP and of HA-TPRKB and c-Myc TP53RK, respectively. In addition, c-Myc TP53RK copurified with HA-OSGEP upon anti-c-Myc immunopurification, although this interaction was not detected in the reciprocoal experiment, raising the possibility that the c-Myc epitope was occluded and/or that binding of the antibody to the epitope disrupts the interaction. Taken together, these experiments established that human EKC has the linear configuration LAGE3-OSGEP-TP53RK-TPRKB, consistent with the three-dimensional structures of archael EKC orthologues that were recently reported [28].

**Figure 4:**
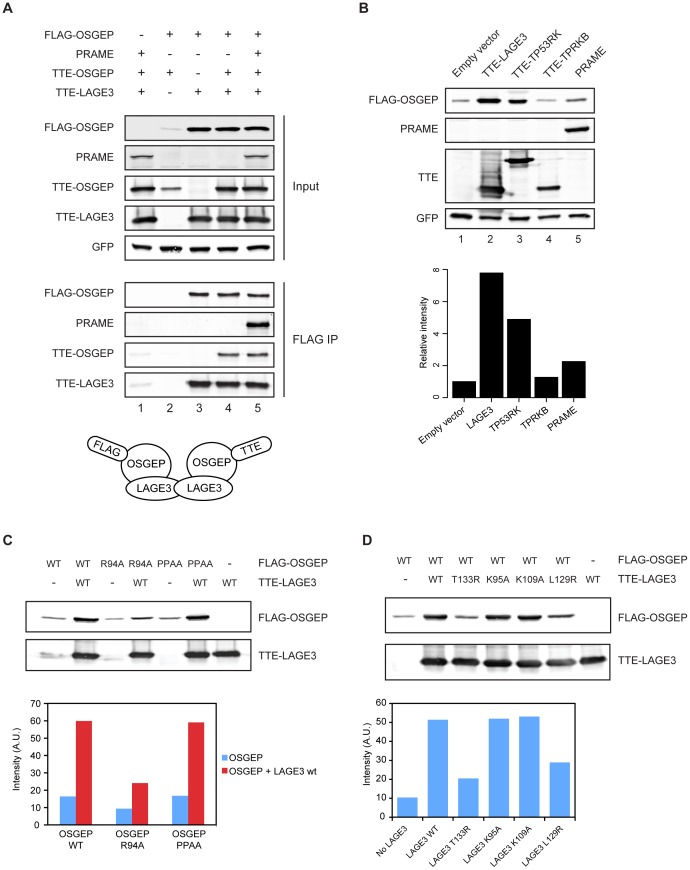
OSGEP protein levels are modulated by protein-protein interactions. (A) Multiple OSGEP moieties are present in the same complex. Constructs expressing the indicated proteins were transiently transfected in 293T cells. Total cell lysates and FLAG eluates were analyzed by western blotting. A plasmid expressing GFP was cotransfected to control for the transfection efficiency. (B) OSGEP levels are affected by protein-protein interactions. 293T cells were transfected with constructs expressing FLAG-OSGEP and the proteins indicated. The bar graph shows FLAG-OSGEP levels quantified by immunoblot with the Odyssey system (values are the average of two independent experiments). A plasmid expressing GFP was cotransfected to control for the transfection efficiency. (C) and (D) LAGE3-OSGEP interface mutants decrease OSGEP protein levels. Transient transfections in 293T cells with the mutant constructs indicated and immunoblot by Odyssey of total cell lysates. Graphs report intensities of FLAG-OSGEP quantified with Odyssey (A.U., arbitrary units).

Interestingly, Mao et al. reported that archael Pcc1, the orthologue of human LAGE3, forms highly interdigitated homodimers and hypothesized that dimerization of Pcc1 might support dimerization of the full EKC complex. Moreover, the crystal structures indicated that one moiety of Pcc1 could bind only one moiety of Kae1. We tested the dimerization properties of human EKC using combinations of different epitope tags in transient transfection experiments. As shown in figure 4A, FLAG-OSGEP coimmunoprecipitated TTE-OSGEP when LAGE3 was cotransfected (lane 4), establishing that multiple OSGEP moieties are present in the same biochemical complex. Importantly, the concomitant expression of PRAME did not affect these dimerization properties (Fig. 4A, compare lanes 4 and 5). Taking into consideration the structural information available for archaeal EKC, our data suggest the formation of a tetrameric LAGE3-OSGEP assembly (Fig. 4A).

In the same experiments, we noticed that OSGEP levels were significantly lower in the absence of LAGE3 (Fig. 4A, compare lanes 2 and 4), suggesting that the stability of OSGEP could be critically dependent on interactions with other proteins. To address this hypothesis, we transfected FLAG-OSGEP together with each of the other EKC subunits or PRAME. Fig. 4B shows that LAGE3, TP53RK and PRAME could all enhance the stability of OSGEP. TPRKB did not alter OSGEP levels, consistent with the absence of a direct interaction between these two proteins. Interestingly, LAGE3 exerted the highest positive effect, which correlates with the possibility to form tetrameric LAGE3-OSGEP complexes, while TP53RK can bind only one moiety of OSGEP. The finding that PRAME had a relatively modest effect on OSGEP levels might reflect a less stable interaction between OSGEP and PRAME, than between OSGEP and TP53RK.

We investigated further the effect of LAGE3 on the protein levels of OSGEP in transient transfection experiments with mutant constructs. Based on the crystallographic structures of archael EKC [28], we introduced point mutations in aminoacid residues predicted to be involved in the protein-protein interactions between OSGEP and LAGE3: R94A in OSGEP, and L129R or T133R in LAGE3. Additionally, we generated OSGEP mutant P35A/P36A (referred to as PPAA) and LAGE3 mutants K95A and K109A, which are not predicted to contribute to the OSGEP-LAGE3 interaction. As shown in figure 4C, the OSGEP mutant R94A showed lower steady-state levels, which could not be enhanced by co-expression of wild-type LAGE3, while OSGEP PPAA behaved like the wild type. Similarly, levels of wild-type OSGEP were not enhanced upon co-expression of either T133R or L129R LAGE3 mutants, while the LAGE3 lysine mutants behaved the same as wild type LAGE3.

Taken together, our data suggests (i) that the human EKC complex is organized in the same way as its archaeal counterpart, (ii) that dimerization of LAGE3 can mediate formation of higher order structures containing at least two molecules of OSGEP, (iii) that formation of these higher order structures is independent of PRAME, and (iv) that the protein levels of OSGEP can be significantly modulated by protein-protein interactions.

### PRAME Bridges Cullin2 Ligases to the EKC Complex

**Figure 5:**
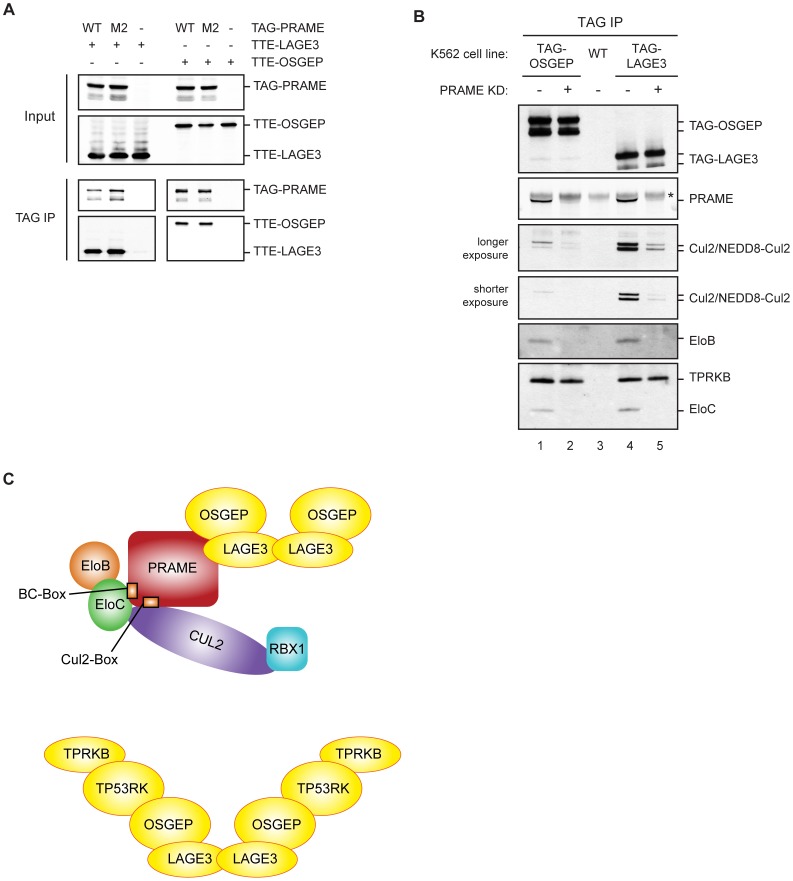
PRAME recruits Cul2-EloBC ligases to EKC. (A) PRAME does not require an intact BC-box to interact with EKC components. Coimmunoprecipitation assays as in Fig.1A with wild type and BC-box mutant M2 PRAME. (B) PRAME bridges Cullin2 ligases to EKC complex. Immunoblot analysis as in Fig. 2 of TAG-OSGEP or TAG-LAGE3 immunoprecipitates with or without knock down of endogenous PRAME. Asterisk indicates heavy chains of the antibodies used in the immunoprecipitation. (C) Models of the protein complexes architecture.

We next studied the role of the Cullin2 complex in the association of PRAME with EKC. Transient transfection experiments showed that the interaction of PRAME with OSGEP and LAGE3 does not require an intact BC-box, since a BC-box defective PRAME (PRAME M2) coimmunoprecipitated OSGEP and LAGE3 as efficiently as wild type PRAME (Fig. 5A).

Next, we purified TAG-LAGE3 protein complexes from HeLaS3 cells, which express very low to undetectable levels of endogenous PRAME. As expected, all EKC subunits copurified with TAG-LAGE3, while no peptides were identified for PRAME (Table 2). Importantly, components of Cullin2 ligases did not copurify with LAGE3 in these cells. The abundance of EKC subunits was similar in the eluates from K562-TAG-LAGE3 and HeLaS3-TAG-LAGE3 cells, consistent with similar protein levels in the two cell lines.

These results, together with the finding that TAG-OSGEP and TAG-LAGE3 associate with endogenous PRAME and Cullin2 ligase components in K562 cells, suggested that PRAME could be involved in recruiting a Cullin2 ligase to the EKC complex.

To test this hypothesis, we knocked down endogenous PRAME with stable transfection of retroviral constructs in K562-TAG-OSGEP and K562-TAG-LAGE3 cell lines and performed immunoprecipitations. Figure 5B clearly shows that downregulation of PRAME significantly reduced the interaction between each EKC subunit and Cul2, EloB and EloC, establishing that PRAME is required for these interactions. As expected, the interaction of TAG-OSGEP and TAG-LAGE3 with TPRKB was not affected by PRAME knockdown. Notably, we found that the level of Cul2 detected in TAG-OSGEP eluates was substantially lower than in TAG-LAGE3 eluates (Fig. 5B, lanes 1 and 4), despite similar amounts of EloB and EloC. These results are similar to the previous comparison between TAG-OSGEP and TAG-PRAME eluates (Fig. 2, lanes 4 and 6).

Taken together, our data reveal that PRAME can bridge Cullin2 ligases to the EKC complex (Fig. 5C).

### Cul2-PRAME Ligases and EKC Subunits Co-localize to Active Promoters

The yeast orthologues of OSGEP (Kae1p) and LAGE3 (Pcc1p) have been reported to associate with the promoters and transcribed regions of several genes in a transcription-dependent manner [17]. Genome-wide chromatin immunoprecipitation experiments revealed that PRAME and Cullin2 complex components are specifically enriched at transcriptionally active promoters that are also bound by the transcription factor NFY, and at nearby enhancers [15].

**Figure 6:**
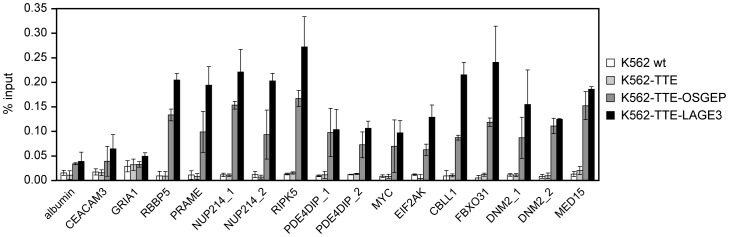
OSGEP and LAGE3 are present at PRAME-bound promoters on chromatin. ChIP-qPCR experiments using BB2 antibody were performed on K562 cells lines stably expressing TTE-OSGEP or TTE-LAGE3. As control for specificity, ChIPs were performed on the parental cells (wt) and on cells transduced with the vector expressing the tag only (K562-TTE) using a panel of primer sets for PRAME biding sites. Values are expressed as mean recoveries ± standard deviation from three independent experiments.

In the absence of validated ChIP-grade antibodies against EKC subunits, we generated K562 cell lines stably expressing the proteins of interest fused to the TTE epitope tag (TY1-TY1-ER), which can be efficiently and specifically used in ChIP-qPCR experiments [15]. To determine whether human OSGEP and LAGE3 are present on chromatin, we performed ChIP assays with the BB2 antibody (which recognizes the TY1 tag) and tested a panel of PRAME-bound promoters. The results in figure 6 clearly show that all PRAME-bound promoters tested are efficiently recovered in both K562-TTE-OSGEP and K562-TTE-LAGE3 cells, but not in parental K562 cells or control K562-TTE cells.

Our genomic experiments extend the biochemical interactions to the chromatin environment, establishing that PRAME and EKC subunits associate with the same promoter regions.

## Discussion

We recently reported the first purifications of PRAME-containing protein complexes by epitope-tagged immunoprecipitations and mass spectrometry, which revealed that PRAME is a BC-box substrate receptor component of Cullin2-based E3 ubiquitin ligases [15]. In the present study, we identified novel interactions of Cul2-PRAME ligases with the human EKC/KEOPS complex, we discovered that PRAME recruits ligase components to EKC, and we showed that these proteins associate with the same promoter regions, strongly suggesting a functional link between these complexes in the same pathway.

EKC is an ancient and extremely conserved complex with orthologues in Archaea and Eukarya. It has been implicated in telomere maintenance, transcriptional regulation, and t^6^A modification of tRNAs in yeast [16], [17], [20]–[22]. Our interaction data support a linear architecture LAGE3-OSGEP-TP53RK-TPRKB for the human EKC complex and dimerization around the LAGE3 subunit, with two OSGEP moieties per complex. Our results are fully consistent with recently reported crystal structures of archaeal EKC proteins [28].

Consistent with the notion that all subunits contribute to the activity of the complex, yeast EKC was reported to constitute a stable entity. Downey et al. reported that deletion of either Bud32p, Cgi121p, or Gon7p resulted in shorter telomeres. An independent study found that Pcc1p was required for efficient transcription of several yeast genes induced by pheromones or galactose, and that deletion of Pcc1 inhibited the recruitment of the SAGA and Mediator co-activators [17]. Dimerization of the Pcc1p and Kae1p subunits was found to be necessary for cell viability [28], and an intact interaction surface between Kae1p and Bud32p was required for both the transcriptional and telomere maintenance functions [29]. Most recently, the yeast EKC complex was shown to be essential for a universal chemical modification of tRNAs, called threonyl carbamoyl adenosine (t^6^A) [20]–[22]. This modification occurs at position 37 of tRNAs decoding ANN codons, including the first ATG of all protein-coding mRNAs, and is required for efficient translation [23]. The t^6^A modification activity was dependent on Pcc1p, the ATPase activity of Kae1p, and the kinase activity of Bud32p, but not on Cgi121p [22].

In our experiments, all human EKC orthologues co-purified together and with similar abundance both in K562 and HeLaS3 cells, demonstrating for the first time that a complete and stable EKC complex is present in human cells. However, we consistently found that PRAME complexes contained primarily OSGEP and LAGE3, and very low or undetectable levels of the other two subunits TP53RK and TPRKB. This might reflect a PRAME-mediated disruption of an otherwise stable EKC complex, or a preferential binding of PRAME to an already existing (and possibly dynamic) EKC submodule that was not previously detected in yeast. It is tempting to speculate that PRAME could mediate ubiquitination of EKC components via recruitment of the Cul2 E3 ligase complex. In an attempt to test this hypothesis, we performed ubiquitination assays upon transient transfections in HEK293 cells in presence or absence of the proteasome inhibitor MG132, and in vitro assays with purified proteins. As expected for BC-box proteins, we detected polyubiquitin chains associated with PRAME itself. However, the high background signals in these assays did not allow us to detect a putative ubiquitination of EKC subunits. Hence, our assays could not rule out that EKC might be ubiquitination targets of Cul2-PRAME, and more efforts are needed to test this hypothesis experimentally.

Interestingly, dynamic regulation of EKC components has been reported: the kinase activity of Bud32p was repressed by association with Kae1p [29], but promoted by binding to Cgi121p [28]. Moreover, we found that the protein levels of OSGEP can be significantly modulated by association with other proteins (Fig. 4), with LAGE3 exerting the most pronounced positive effect, followed by TP53RK and PRAME. Interestingly, the effect of LAGE3 correlates with the formation of tetrameric complexes in which an LAGE3 dimer binds two OSGEP moieties (Fig. 4A), which are likely not in direct contact with each other [28].

The results of our transient transfection experiments and those obtained using the baculovirus system suggested (i) that PRAME is able to interact independently with LAGE3 and OSGEP and (ii) that LAGE3 enhances the association of OSGEP with PRAME. Considering the dimerization properties of the LAGE3-OSGEP submodule, we therefore considered the possibility that multiple PRAME moieties might be present in the same protein complex. In this case, both epitope-tagged PRAME and endogenous PRAME should be present in the same protein complex in K562-TAG-PRAME cells, since the expression level of the two proteins were similar. Contrary to this hypothesis, however, TAG-PRAME did not co-immunoprecipitate endogenous PRAME in our experiments (see Fig. 2 lane 4). Although we cannot formally exclude the possibility that TAG-PRAME might compete with the endogenous protein, our data are most consistent with the idea that there is only one PRAME molecule per complex. Notably, our data suggest that the interaction between PRAME and LAGE3-OSGEP is very stable, since it was efficiently detected in all assays and despite the long incubations times in our protein-complex purification protocols. However, we cannot exclude that high levels of PRAME might be required for the interactions to take place in cells. Taking into account the published crystal structures of archaeal EKC, we propose the models depicted in Figure 5C for the architecture of the human EKC complex and the relationship between EKC and Cul2-PRAME ligases. Further experiments are needed to confirm the stoichiometry of PRAME-EKC complexes, and to characterize the interaction surfaces involved.

Besides the functional experiments in yeast, no information is available yet about EKC functions in other organisms. At present, only studies focusing on single subunits of the human complex have been reported. LAGE3 appears to be ubiquitously expressed, while the two closely related LAGE1 and LAGE2 are cancer-testis antigens with high expression confined to healthy testis and cancer tissues [24]. Interestingly, expression of the N-terminal domain of LAGE3 fused to the C-terminus of Pcc1p could partially rescue the growth defects of a Pcc1 N-terminal mutant yeast strain, while full length *LAGE3* could not substitute for Pcc1 [17]. The *OSGEP* gene was found expressed in all somatic tissues tested and it lies in a head-to-head orientation with the tightly regulated *APEX* gene, which encodes a multifunctional DNA repair enzyme [30]. Analyses of their bidirectional promoter identified a CCAAT box crucial for *OSGEP* transcription. Futhermore, *OSGEP* could partially rescue the growth defects of the yeast *kae1*Δ mutant [17], underlining degrees of functional conservation. Human *TP53RK* was first cloned by cDNA subtraction as an interleukin-2 upregulated gene in cytotoxic T-cells, transcripts were detected in a number of normal tissues and cancer cell lines, and it was shown to phosphorylate the human oncoprotein p53 at Serine 15 [31]. Recombinant TP53RK expressed in bacteria was found catalytically inactive, but incubation with COS-7 cell lysates was sufficient to activate the enzymatic activity, suggesting the need for activators and potentially protein interactors. The kinase Akt/PKB was found to play a role in the activation of TP53RK through phosphorylation of the Ser250 residue [32]. Underscoring a high level of functional homology, TP53RK could partially rescue the growth phenotype of Bud32 deletion in yeast, and Bud32 could interact with and phosphorylate human p53 in vitro [33], although yeast does not possess a *bone fide* p53 homologue. Human *TPRKB* was first isolated by a two-hybrid screen using TP53RK as a bait, and was suggested to inhibit the binding of TP53RK to p53 [34]. Expression of *TP53RK* was detected in the high-PRAME expressing cell line K562 as well as in the low-PRAME expressing cell line KG1 [34]. Gene expression data obtained from the GeneSapiens database further suggests that EKC subunits are expressed in most if not all human tissues (Figures S2, S3, S4).

Considering the extreme conservation of EKC components during evolution, it can be expected that at least some of the functions and pathways already identified for this complex in yeast could be valid for human cells as well. However, it cannot be excluded that human EKC acquired additional, yet to be identified functions during evolution. The absence of efficient knock-down or knock-out models have so far hindered advanced functional analysis of EKC in higher eukaryotes. Future studies will have to address these issues, and in particular to understand the connections between apparently different functions ascribed to this complex, like phosphorylation of the p53 oncogene, chemical modification of tRNAs, and physical association with promoter regions.

Kae1p and Pcc1p have been found to associate with active chromatin and to regulate expression of some of the yeast promoters to which they are recruited [17]. A *pcc1-4* mutation prevented activation of *GAL1* by impairing recruitment of the SAGA histone acetyltransferase and mediator without affecting recruitment of the activator Gal4p. In contrast, while Pcc1p was recruited to the constitutively active *PMA1* and the heat-shock inducible *HSP104* genes in a transcription-dependent manner, transcription of these genes was not affected in the *pcc1-4* mutant.

Our ChIP assays revealed that LAGE3 and OSGEP are also present on chromatin and, in particular, that they colocalize with PRAME at many of its target sites, consistent with our protein-protein interaction data. Since neither PRAME nor EKC proteins possess known DNA-binding domains, it is not clear whether they can bind DNA directly. Genome-wide ChIP-seq experiments have recently revealed that PRAME associates with transcriptionally active NFY-regulated promoters and nearby enhancers [15]. Therefore, the transcription factor NFY might play a direct or indirect role in the targeting of a Cul2-PRAME-EKC complex to chromatin. It is also not clear whether there is a hierarchy in the association of PRAME and EKC proteins with chromatin. In order to address this point, we established stable cell lines with downregulation of endogenous PRAME using the published retroviral pRetroSuper system in either K562 wild type or K562-TTE-LAGE3 cells and we performed ChIP experiments. Unfortunately, despite a 70% downregulation of PRAME mRNA levels, our preliminary results did not support a solid conclusion on the hierarchy of binding to chromatin for PRAME and EKC. More experiments, and likely in a different cell and knockdown system, are required to address this point.

Importantly, EKC proteins have an ancient evolutionary origin and seem to be ubiquitously expressed in adult tissues, while PRAME appeared more recently in evolution, and its expression seems to be tightly confined to particular developmental stages and tissues and to be particularly deregulated in oncogenesis. These characteristics are consistent with a model in which EKC can associate with chromatin independently of PRAME. Only when *PRAME* is expressed would a Cul2-PRAME ubiquitin ligase be recruited to chromatin by virtue of specific protein-protein interactions between LAGE3-OSGEP and PRAME.

In an attempt to study the putative function of EKC subunits on target gene transcription, we established K562 cells with stable downregulation of OSGEP or LAGE3 using pRetroSuper vectors and performed genome-wide mRNA-seq analyses. Despite a 50–80% knockdown efficiency for OSGEP and LAGE3, we did not detect genes with significant expression changes when compared to a GFP knockdown control. Our inconclusive data could of course be the result of inefficient downregulation of the target genes. Although the K562 cell line was successfully used to dissect the protein-protein interaction network, we cannot exclude that this cell line might not be suitable for the study of the finer putative chromatin functions and signaling of EKC and PRAME.

The development of ChIP-grade antibodies against EKC components will be a crucial step to address important questions as to whether PRAME target sites are a subset of EKC sites (or viceversa), and to address the functional relationships between EKC, Cul2-PRAME ligases and NFY on transcriptional regulation of target genes, and potentially other pathways like the recently described modification of tRNAs.

Taken together, our data reveal a novel link between the human oncoprotein PRAME and the ancient EKC complex. Our data clearly show that PRAME can recruit Cullin2 ubiquitin ligases to EKC, and that these complexes associate with the same genomic regions, supporting a functional link in the same pathways. Our results add a new twist to both PRAME and EKC biology and provide an important contribution to understanding the molecular mechanisms in which these proteins are active, both in healthy and cancer cells.

## Materials and Methods

### Cell Culture and Stable Cell Lines

HEK293T cells were cultured in DMEM and K562 and HeLaS3 in RPMI medium (Gibco, Invitrogen) at 37°C in 5% CO_2_; cell lines were obtained from ATCC. Both media were supplemented with 5% Glutamax, 10% fetal bovine serum, 100 U/ml of penicillin, 100 µg/ml of streptomycin (Gibco, Invitrogen). K562 and HeLaS3 stable cell lines were generated with retroviral constructs as described [15]. Transient transfections were performed with Lipofectamine2000 (Invitrogen).

### Antibodies and Western Blot

Mouse monoclonal HA-12CA5 and Myc-9E11 were produced in-house from hybridoma cultures, PRAME antibodies were previously described [15]. Commercial antibodies used were: mouse monoclonal FLAG-M2 (Sigma), c-Myc monoclonal (Roche Applied Science), rabbit HA (Bethyl Laboratories), mouse monoclonal HA.11 (Covance MMS-101P), rabbit Cul2 (Zymed Laboratories 51-1890), mouse ElonginC (BD Transduction Laboratories 610760), goat ElonginB (Santa Cruz P-16, sc-23407), mouse TPRKB (Abcam ab68245), mouse BB2 against TY1 tag (Diagenode), GFP (Santa Cruz). Proteins were separated by conventional SDS-PAGE or NuPAGE 4–12% Bis-Tris gels (Invitrogen) run with MES buffer. Western blots were visualized by ECL (GE Healthcare) or Odyssey (LiCor) for quantification.

### Plasmids and Cloning

A modified version of the retroviral vector LZRS(Zeo) with an improved MCS was made by ligation of a synthetic oligo to generate LZRSn(Zeo). The sequence coding for the StrepII-TEV-MYC-3xHA tag was excised with BamHI and NotI from pcDNA5-TAG-PRAME and subcloned in LZRSn(Zeo) to generate LZRSn-TAG, where the EcoRI site is in the same frame as in the pTTE retroviral vector [15].

The TTE cassette was subcloned from pTTE [15] to pcDNA3.1(+) using BamHI and XhoI sites to generate pcDNA-TTE for transient transfections.

Full-length human OSGEP was amplified by PCR from cDNA prepared from K562 cells to generate a NotI-EcoRI-OSGEP-SalI fragment, which was cloned into pFLAG-CMV2 (pFLAG-OSGEP). OSGEP was subsequently subcloned as an EcoRI-SalI fragment into the EcoRI and XhoI sites of pTTE and pcDNA-TTE, to generate pTTE-OSGEP and pcDNA-TTE-OSGEP, respectively. OSGEP was cloned as a NotI-SalI fragment downstream the TAG in LZRSn(Zeo), generating LZRSn-TAG-OSGEP.

LAGE3 CDS was obtained from Invitrogen, PCR amplified to introduce EcoRI and XhoI sites, and subcloned in the same sites of pTTE, pcDNA-TTE, and LZRSn-TAG-LAGE3.

Full-length TP53RK/PRPK and TPRKB were amplified by PCR from cDNA of K562 cells and subcloned as EcoRI and XhoI fragments to generate all constructs used.

Mutagenesis to generate OSGEP and LAGE3 mutants was performed using the QuikChange II XL site-directed mutagenesis kit (Stratagene) according to the manufacturer’s instructions.

All constructs were checked by sequencing.

### Purification of Protein Complexes and Mass Spectrometry Analysis

Protein complex purifications and mass spectrometry were performed from nuclear extracts (unless otherwise indicated) essentially as described [15]. For HeLaS3 cells the protein extraction protocol was performed with a tighter douncer (pestle B). The quality of cytoplasmic and nuclear extractions were tested by immunoblotting of equivalent volume fractions for alpha tubulin (cytoplasmic marker) and HDAC2 (nuclear marker).

For TAG-PRAME purifications with MYC antibody, about 10 ml of protein extracts (∼100 mg) were incubated with 50 µl (MYC1 experiment) or 600 µl (MYC2 experiment) of crosslinked MYC beads and peptide elution was performed. For protein complex purifications with HA antibody, about 5–6 ml of protein extracts were incubated with 600 µl HA-12CA5 crosslinked beads and acidic glycine (pH 2.9) was used for elution.

The data analysis steps of peptide-to-protein remapping and emPAI calculation were automated with a PERL script to generate output tables of unique protein IDs with peptide frequencies and emPAI values for each samples (see Protocol S1 and Analysis Script S1). The protein list was manually filtered to discard proteins identified in the control samples (false positives).

### Expression of Recombinant Proteins in Sf21 Insect Cells

cDNAs encoding wild type or mutant PRAME, OSGEP, LAGE3, TP53RK and TPRKB were subcloned into pBAcPAK 8 and recombinant baculoviruses were generated. SF21 cells were cultured at 27°C in SF-900 medium supplemented with 10% fetal bovine serum. SF21 cells were co-infected with the recombinant baculoviruses indicated in the figures. 50 hours after infection, cells were collected, lysed, and processed as described [35].

### ChIP and qPCR

Chromatin harvests, ChIPs and qPCR analyses were performed as previously described [15]. The BB2 antibody recognizing the TY1 epitope (Diagenode) was used to ChIP TTE-tagged proteins from K562 stable cell lines.

## Supporting Information

Figure S1 **Detailed expression profiles of** ***MAG*** **,** ***TBP*** **,** ***CUL2*** **, and** ***PRAME*** **from the GeneSapiens database.** Normalized relative gene expression levels in healthy (green) and cancer (red) tissues are plotted as boxplots. *MAG* and *TBP* are shown as references: *MAG* is a known neuronal marker gene and shows an expression profile that is highly specific for the central nervous system. On the contrary, *TBP* is a ubiquitously expressed gene. Similary to *TBP*, the expression profile of *CUL2* indicates ubiquitous expression in both healthy and cancer tissues. On the contrary, *PRAME* is expressed mainly in the healthy testis, and is upregulated in a number of cancers.
(TIF)

Figure S2 **Detailed expression profiles of EKC subunits from the GeneSapiens database.** Normalized relative gene expression levels in healthy (green) and cancer (red) tissues are plotted as boxplots. The expression profiles of *LAGE3*, *OSGEP*, *TPRKB*, and *TP53RK* indicate expression in all or most healthy and cancer tissues.
(TIF)

Figure S3 **Gene expression correlation plots between** ***PRAME*** **and subunits of Cul2 ligases or the EKC complex.** Co-expression plots were generated from the GeneSapiens database for *PRAME* and *CUL2* or each of the EKC subunits. Correlations plots are shown for healthy tissues (A), and cancer tissues (B). The plots indicate that the expression of the genes tested do not correlate linearly. In particular, a large number of samples are characterized by low or no *PRAME* expression, while *CUL2* and EKC subunits are expressed at higher levels.
(TIF)

Figure S4 **Gene expression correlation plots between subunits of Cul2 ligases and subunits of the EKC complex.** Co-expression plots were generated from the GeneSapiens database for *CUL2* and *TCEB1*, and couples of EKC subunits. Correlations plots are shown for healthy tissues (A), and cancer tissues (B).
(TIF)

Protocol S1 **Detailed protocol for comparative analyses of mass spectrometry data.**
(DOC)

Analysis Script S1 **PERL script mapPep2Prot_v0.3.pl for analysis of mass spectrometry data.**
(PL) Click here for additional data file.
