# Supplementary figures and images for: The Human EKC/KEOPS Complex Is Recruited to Cullin2 Ubiquitin Ligases by the Human Tumour Antigen PRAME
Source: PLoS One. 2012 Aug 13;7(8):e42822. doi: 10.1371/journal.pone.0042822 (PMC3418287; doi:10.1371/journal.pone.0042822)

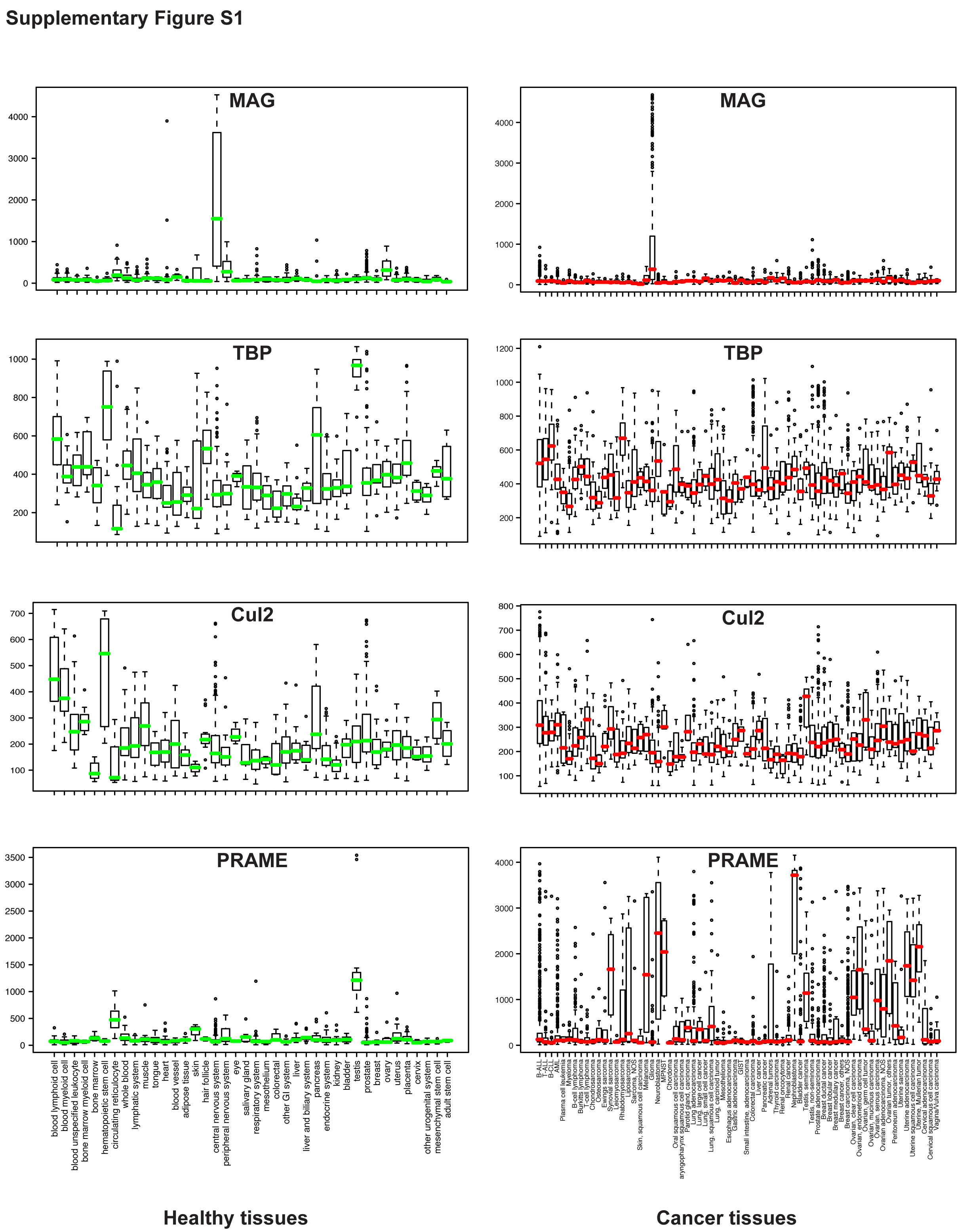

Supplement: Figure S1 — Detailed expression profiles of MAG , TBP , CUL2 , and PRAME from the GeneSapiens database. Normalized relative gene expression levels in healthy (green) and cancer (red) tissues are plotted as boxplots. MAG and TBP are shown as references: MAG is a known neuronal marker gene and shows an expression profile that is highly specific for the central nervous system. On the contrary, TBP is a ubiquitously expressed gene. Similary to TBP, the expression profile of CUL2 indicates ubiquitous expression in both healthy and cancer tissues. On the contrary, PRAME is expressed mainly in the healthy testis, and is upregulated in a number of cancers. (TIF) [file pone.0042822.s001.tif]

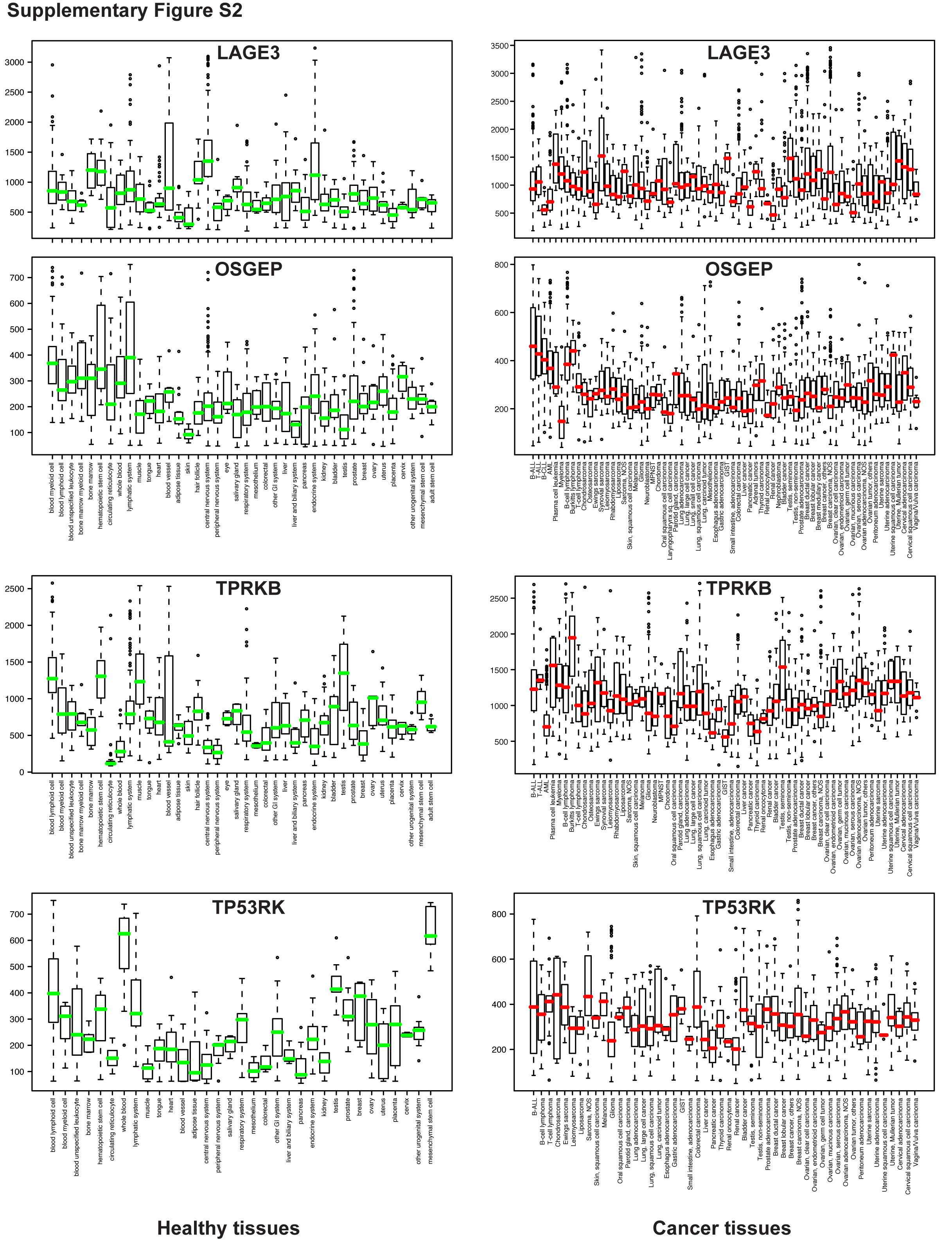

Supplement: Figure S2 — Detailed expression profiles of EKC subunits from the GeneSapiens database. Normalized relative gene expression levels in healthy (green) and cancer (red) tissues are plotted as boxplots. The expression profiles of LAGE3, OSGEP, TPRKB, and TP53RK indicate expression in all or most healthy and cancer tissues. (TIF) [file pone.0042822.s002.tif]

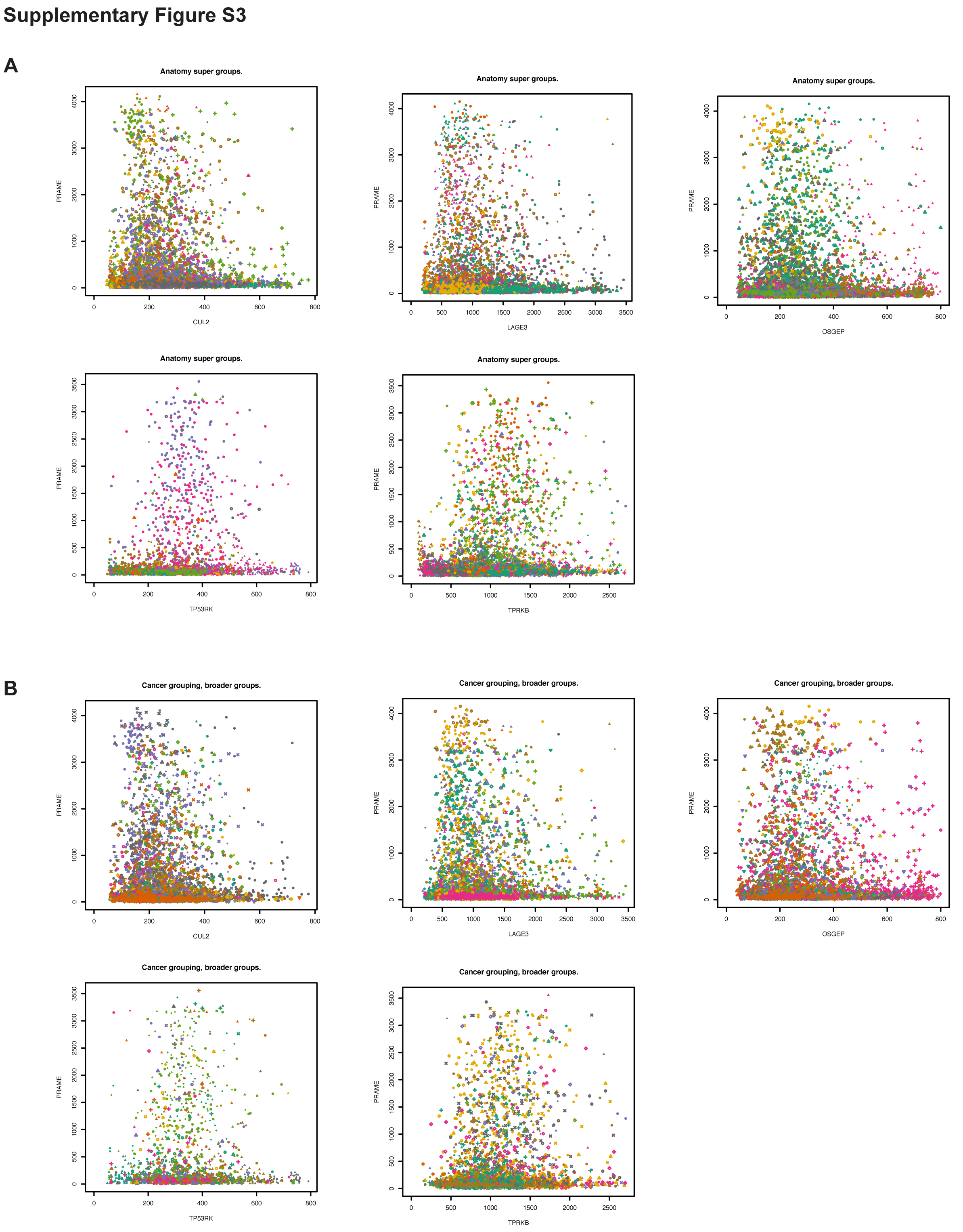

Supplement: Figure S3 — Gene expression correlation plots between PRAME and subunits of Cul2 ligases or the EKC complex. Co-expression plots were generated from the GeneSapiens database for PRAME and CUL2 or each of the EKC subunits. Correlations plots are shown for healthy tissues (A), and cancer tissues (B). The plots indicate that the expression of the genes tested do not correlate linearly. In particular, a large number of samples are characterized by low or no PRAME expression, while CUL2 and EKC subunits are expressed at higher levels. (TIF) [file pone.0042822.s003.tif]

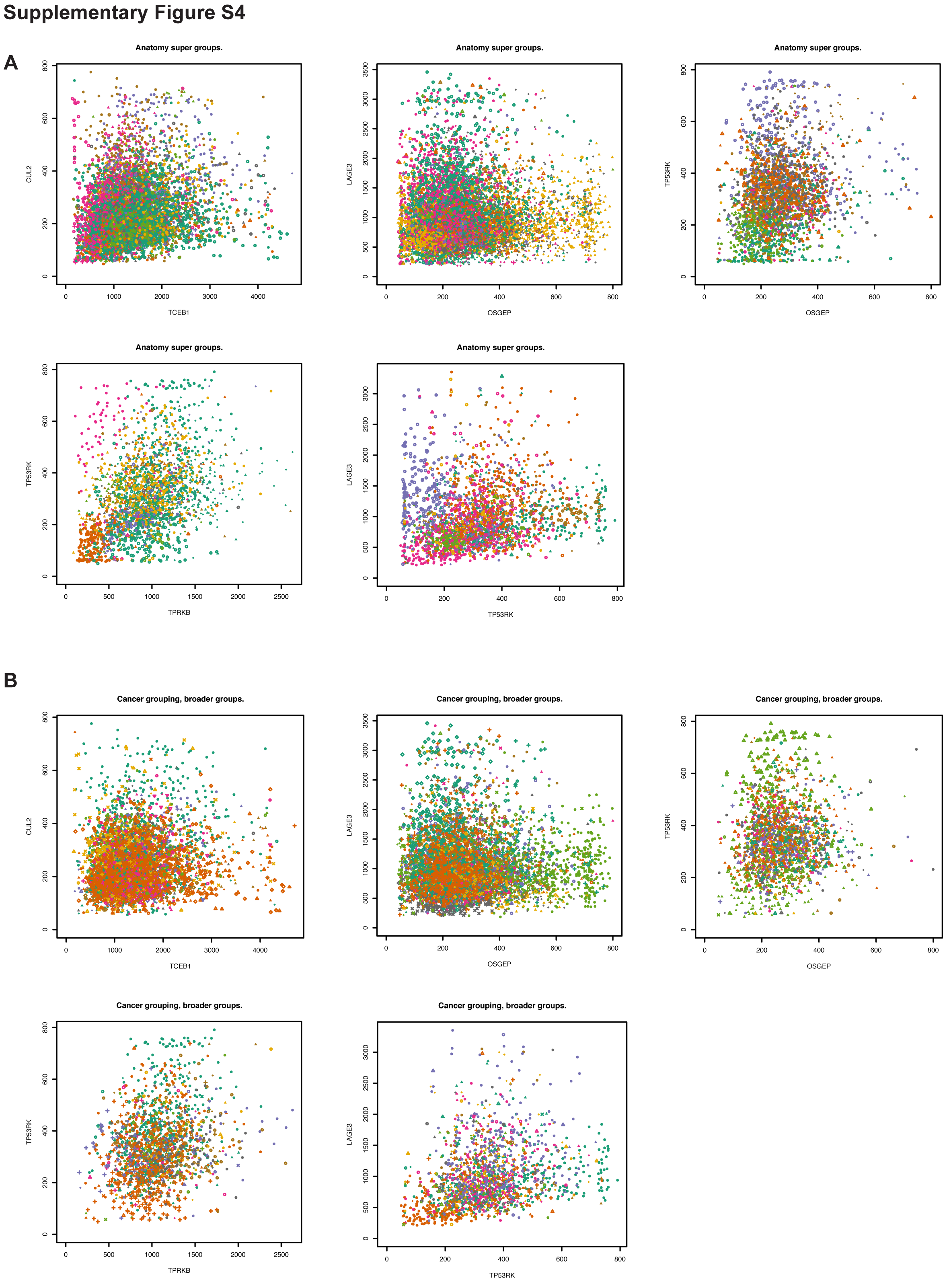

Supplement: Figure S4 — Gene expression correlation plots between subunits of Cul2 ligases and subunits of the EKC complex. Co-expression plots were generated from the GeneSapiens database for CUL2 and TCEB1, and couples of EKC subunits. Correlations plots are shown for healthy tissues (A), and cancer tissues (B). (TIF) [file pone.0042822.s004.tif]
