# Supplementary material for: The Human EKC/KEOPS Complex Is Recruited to Cullin2 Ubiquitin Ligases by the Human Tumour Antigen PRAME
Source: PLoS One. 2012 Aug 13;7(8):e42822. doi: 10.1371/journal.pone.0042822 (PMC3418287; doi:10.1371/journal.pone.0042822)
Supplement: Protocol S1 — Detailed protocol for comparative analyses of mass spectrometry data. (DOC) [file pone.0042822.s005.doc]

**SUPPORTING INFORMATION**

Costessi et al. - The Human EKC/KEOPS Complex Is Recruited to Cullin2 Ubiquitin Ligases by the Human Tumour Antigen PRAME

**SUPPLEMENTARY PROTOCOL S1**

DETAILED PROTOCOL FOR COMPARATIVE ANALYSES OF MASS SPECTROMETRY DATA

A. Introduction and scope

Mass spectrometry analyses are often performed using large and comprehensive databases of protein sequences (i.e. the IPI database, http://www.ebi.ac.uk/IPI/) because this gives the highest chance that peptides measured in an experiment are assigned to a protein sequence in the database. On the contrary, the use of highly curated databases that contain only well-annotated sequences (i.e. Swiss-Prot) can lead to missing novel uncharacterized proteins.

Importantly, comprehensive databases have the disadvantage of being to some extent intrinsically redundant: numerous protein sequences in the database differ from each other in just one or few amino acid residues (i.e. truncated sequences, splicing variants, non-synonimous SNPs, etc). This often leads to a peptide matching more than one protein ID in the database, causing a dilemma of which protein ID to choose. In our experience with MASCOT searches we have noticed that if a peptide matches multiple protein IDs, a "random" assignment takes place and one of the matching protein IDs “at random” is given in the results. Notably, the protein ID displayed in the results for a certain peptide is often different when performing different analyses. This makes it impossible to perform a simple comparison of protein IDs from different experiments, and a detailed analysis is therefore very laborious and time consuming.

To address these issues, we implemented an analysis pipeline that addresses the redundancy of peptide-to-protein matching. Our pipeline makes use of two freeware programs (Protein Coverage Summarizer and Protein Digest Simulator) and an in-house developed PERL script (mapPep2Prot_v0.3.pl) to rapidly generate comprehensive tables of protein IDs with peptide frequencies and emPAI values that can be directly compared between different samples.

The algorithm was developed in Henk Stunnenberg's lab (Dept. Molecular Biology, NCMLS, Radboud University Nijmegen, The Netherlands) by Adalberto Costessi and was scripted in PERL by Blaise Alako. The approach was developed for searches of human peptides against the IPI database, but can be applied to other databases with minor modifications to the ranking scheme in the script code. The algorithm is based on the MS analysis strategy published in: Lasonder E. et al (2008) *Proteomic profiling of Plasmodium sporozoite maturation identifies new proteins essential for parasite development and infectivity*. PLoS Pathog.

Detailed information on the script itself (settings, usage) are provided in the manual of the script (see below).

For the purpose of this protocol, “MS dataset” or “MS sample” refers to the MS analysis of one particular biological sample (i.e. IP against a protein of interest). This protocol is used to analyse several MS datasets (i.e. several MS samples) together.

Before starting the analysis

First of all, raw mass spec data are used in a MASCOT search against the IPI db. The resulting html files are parsed with MSQuant to generate unique ranked peptide lists for each sample. These lists are further filtered for absolute calibrated mass error <5, delta score >5, and MASCOT score ≥ 20, resulting in a high-confidence peptide list for each sample.

Materials required for the comparative MS analysis:

1. High-confidence peptide lists for each sample to analyse
2. A full version of the IPI human db used in the MASCOT search (in FASTA format)
3. A Windows computer and the freeware programs:
   1. Protein Coverage Summariser (<http://omics.pnl.gov/software/ProteinCoverageSummarizer.php>)
   2. Protein Digest Simulator (<http://omics.pnl.gov/software/ProteinDigestionSimulator.php>; this is only required to generate emPAI values)
4. A UNIX environment with PERL
5. Our PERL script: mapPep2Prot_v0.3.pl

Calculation of the number of “theoretically observable” peptides for emPAI analysis

The exponentially modified Protein Abundance Index (emPAI) provides a measure of abundance of a protein in a sample. (Ishihama Y et al. (2005) *Exponentially modified protein abundance index (emPAI) for estimation of absolute protein amount in proteomics by the number of sequenced peptides per protein*. Mol Cell Proteomics 4: 1265–1272). The emPAI value is calculated as 10^PAI–1^ (PAI = observed peptides/observable peptides). The number of observable peptides can be calculated for all entries in a database based on the technical specifications of the mass spectrometry used for the experiments.

We used Protein Digest Simulator to perform an *in silico* digestion of the entire IPI Db (v3.37) and to subsequently count the number of peptides we could theoretically observe in our experimental conditions.

For the *in silico* digestions, it is important to use the same database version used in the MASCOT searches, and the same enzymes used in the mass spec sample preparation (in our case IPI v 3.37 and trypsin). The settings of the digestion need to take into account the technical specs of the mass spectrometer used.

We set the following parameters in Protein Digest Simulator:

- In silico digest of all proteins (selected)
- Fully tryptic (KR no P)
- Min 600
- Max 4000
- Generate unique ID values (selected)
- Min residue count: 0
- Max missed cleavages: 0
- Computer protein mass (selected)
- Include X residues (selected)
- NB: do not compute pI

File format options:

- >
- space
- Protein Name, Description, Sequence

Output options: keep the option “Include prefix and suffix residues for the sequences” deselected.

The output file (ipi.HUMAN.v3.37_trypsin_digested_digested_Mass600to4000.txt) is a list of all the theoretically observable peptides for the proteins in the db. The following step is to count the total number of peptides for each protein ID. For this we used the following command line to create a file with two tab-delimited columns (# pept; IPI protein ID):

cut -f1 ipi.HUMAN.v3.37_trypsin_digested_digested_Mass600to4000.txt | sort | uniq -c | awk '{print $1 "\t" $2}' > **observablePeptides.IPIhuman3.37.MW_600-4000**

The first line of the file looks like this:

36 IPI:IPI00000001.2|SWISS-PROT:O95793-1|TREMBL:A8K622;Q59F99|ENSEMBL:ENSP00000360922;ENSP00000379466|REFSEQ:NP_059347|H-INV:HIT000329496|VEGA:OTTHUMP00000031233

Note that the IPI db reports also all cross-references for each protein sequence to other databases, as clearly shows above. This file will be used by the PERL script to calculate emPAI values for observed proteins.

B. Protocol for mass spectrometry data analysis

1. Prepare peptide files per sample

For each MS dataset you want to analyse, you need to generate a TAB-delimited file containing the high-confidence score peptides. The file will contain two columns: the first is the amino acid sequence of the peptide, the second column will be the MASCOT score of that peptide. For Windows machines, this file can be generated by copy-paste of the corresponding columns from Excel into Notepad. Give the file a filename that doesn't contain spaces: **SAMPLE_NAME.pep**

Example:

SFSALAELVAAAK 42

YDSQVAEENR 47

2. Prepare total peptide list

Generate a non-redundant list of ALL the peptides from ALL the samples that you are going to analyse. You can paste all the peptide sequences (NB: only the peptide sequences, no MASCOT scores) from each **SAMPLE_NAME.pep** into one Excel file, and make the list non-redundant, so that each sequence will appear only once in the list.

This list can be generated very easily with Unix commands starting from the **SAMPLE_NAME.pep** files:

- Fix new-line sign with PERL command (if needed):
  **for i in *.pep ; do perl -lne '~s/\r/\n/g; print' $i > fix.$i; done**
- For peptide files made in Windows Notepad, there is both \r and \n at the end of a line, so you need this command instead: **for i in *.pep ; do cat $i | tr -d “\r” > fix.$i; done** (otherwise, you get \n \n, so an empty line between two peptides)
- Concatenate all peptides (Mascot scores are still present):
  **for i in fix*; do cat $i >> allpeptides ; done**
- Generate unique peptides list w/o score:

**cut -f1,1 allpeptides | sort | uniq > allpeptides.unique.txt**

Example (8574 unique peptides):

wc -l allpeptides*

17694 allpeptides

8574 allpeptides.unique.txt

3. Re-mapping of the peptides with Protein Coverage Summarizer

Run Protein Coverage Summarizer to re-map the peptides to the same protein database used for the MASCOT searches (in our case IPI human v3.37).

Input:

- **allpeptides.unique.txt** (in this example: 8574 lines)

- **Fasta formatted IPI_human Db** (v3.37)

Settings:

- uncheck "Skip first line in protein input file"

- select: Column Order - Sequence Only

- uncheck "Skip first line in peptide input file"

- Check all the other options (very important!), but uncheck the last two options: "Skip coverage computation" and "Ignore I/L Difference"

Output:

**- allpeptides.unique_ProteinToPeptideMapping.txt**

- **allpeptides.unique_coverage.txt**

NB: rename these two output files, by adding the extension **.prot** to the file names:

**allpeptides.unique_ProteinToPeptideMapping.txt.prot**

**allpeptides.unique_coverage.txt.prot**

----------------------------------------------------------------------

Additional information on the two output files:

**allpeptides.unique_ProteinToPeptideMapping.txt** **[27897 lines]**

This files contains all possible assignments for each peptide to matching IPI proteins in the format:

ProteinID | Peptide Sequence | Residue Start | Residue End

You can count how many peptides are present in the results:

cut -f2 allpeptides.unique_ProteinToPeptideMapping.txt | sort | uniq | wc -l

**8562** (NB: one line is the header; so only 11 peptides were lost in this step in the example, which is a good result)

**allpeptides.unique_coverage.txt [69165 lines]**

This file contains all the data for all the IPI_human proteins in the format:

Protein Name | Percent Coverage | Protein Description | Non Unique Peptide Count | Unique Peptide Count | Protein Residue Count

----------------------------------------------------------------------

4. Generation of comparative tables with PERL script for multiple sample comparison

Run the **PERL script** as described in its manual, choosing the parameters according to the outputs required.

The following files need to be present in the same folder:

- peptide lists per sample (.pep files)
- the two re-mapping output files (.prot files)
- the file containing the observable number of peptides (for emPAI calculation)
- the perl script mapPep2Prot_v0.3.pl

The script should be run like this:

**perl mapPep2Prot_v0.3.pl -pep *.pep -prot *.prot -o peptides_mapping.txt -h -t observablePeptides.IPIhuman3.37.MW_600-4000 -all -md**

This syntax generates a number of comprehensive tables with all unique protein ID and frequencies of peptide/sample as well as emPAI factor per sample.

More detailed information of the script options are found in the script documentation.

5. Results

The results are comprehensive tables of non-redundant, uniquely mapped peptides. These can be manually inspected to apply filters to remove background and false-positives (i.e. proteins identified in the control samples).

**MAPPEP2PROT_V0.3(1) User Contributed Perl Documentation**

**NAME**

sample usage: mapPep2Prot.pl [options...] -pep *.pep -prot *.prot -o

output.txt -h -t observable.peptides.file -all

Where: *.pep [peptide file names with .pep extension]

*.prot [protein related file name with .prot extension]

**DESCRIPTION**

**Algorithm**: This PERL script is designed to help in the analysis of

Mass-Spectrometry interaction data. Some input files have to be gener-

ated with free Windows softwares (see below for details). In general,

two aspects are addressed by the script:

1. assign to each peptide identified by Mass Spec the best protein IPI

ID, solving the recurrent problem that two different MASCOT searches

often assign different protein IDs to the same peptide sequence;

2. calculate emPAI abundance factors for each protein ID for all exper-

imental dataset.

**Assigning the best IPI ID to each peptide** It is very common that a pep-

tide sequence can be matched to multiple proteins (in our case we'll be

talking about IPI IDs, but the script can be used with any protein Db

of course). We want to choose one of these IDs (the right one!) and

use this ID to re-map the experimental peptides lists, and then calcu-

late the number of peptides identified for each protein ID accordingly.

This approach makes sure that one peptide is not matched to different

protein IDs in different peptide lists (different MS runs, or different

experiments), which makes the final data non-redundant and we can com-

pare datasets appropriately.

The criteria applied to choose the "best" Protein ID for a certain pep-

tide sequence are: - first, the protein ID with the highest number of

matching peptides is preferred; - if more than one protein ID satisfy

the first criteria, we choose among those the IPI ID with the most

extensive and reliable cross-reference identifiers. For example,

between an IPI ID that contains only an IPI reference, and another ID

that contains also a Swiss-Prot reference, the second one is preferred.

This is in line with the idea that the most extensively a sequence is

annotated, the most reliable it is, and the more information can be

extracted from the databases about it (GO annotation, pathway, etc.).

In line of implementation, the script uses a weighting scheme biased

toward more curated database identifiers, which means that IDs with

more extensive and reliable identifiers are given a stronger weight.

**mapPep2Prot.pl** expects two sets of input files. Please rename them with

the appropriate extension for easy reference: eg. all the peptide files

end with .pep, the 2 protein-related information end with .prot. The

peptide files are tab-delimited two-columns files where the first col-

umn contains the peptide sequence, and the second column contains the

corresponding Mascot score. The 2 protein-related files are the outputs

generated by the freeware program Protein Coverage Summariser (process

to be described): one contains all the possible matches between each

peptide sequence and the IPI IDs present in the database, the second

file (the biggest in size) contains a lot of information, and is used

here to extract the Description (the name) of each IPI ID.

The "standard" **output** of the script is a table of the experimentally-

derived peptide sequences with their best-match IPI ID as defined

above. This output is printed to screen by default (unless the debug -d

option is selected, see below), and it can be saved to file using the

option **-o**.

By activating the option **-all**, the script will use the best-match IPI

IDs to re-map the input peptide (**.pep**) files, by adding two columns:

the best-match IPI ID, and its description (IPI protein name), and this

information is stored in files with extension **.ext** (for EXTended infor-

mation, and the file name is the same as the original .pep file).

The option **-all** generates also 4 overview tables saved in files that

start with **IPIOccurencePerSample.** that contain a big table with all the

IPI IDs identified by the analysis and in each column the number of

peptides for each ID counted in each file. If the option **-empai** is

active and the file with theoretical peptides is given with **-t**, the ta-

ble will contain also the emPAI values for each ID for each sample

(i.e. for each experimentally-derived peptide list). By default the

output files contain only the IPI ID; specify the option -md **to add the**

**Description of the IPI IDs. By specifying the option -h,** headers will

be printed in the first line of these 4 files.

**SYNOPSIS**

mapPep2Prot.pl [options] -s [peptide files ...] -l [protein files ...]

Options:

-help brief help message

-man full documentation

**OPTIONS AND ARGUMENTS**

-peptide|pep|s

Files containing the peptide and the Matscore -- preferably

called through : -s *.pep.

-protein|prot|l

Files containing the protein to peptide information -- prefer-

ably called through : -l *.prot.

-o|output defaults to screen

File where to store the mapping.

-t|theoretical mandatory

File containing the theoretical peptide occurence per protein

(IPI) id.

-header|h switch-off by default

Add header to the output files

-debug|-d switch-off by default

Dump a data structure of the data being process, detailed

information to track down errors and comprehending what the

program does.

-limit|l default 40

Chunck size of the data to process, this is only if the debug

flag is switched on.

-gm|sum default true

report in a tabular form (matrix-like), the IPI and their

occurence per uploaded peptide ids. The column in this repre-

senting the different sample file and the rows the unambigous

IPI identifiers

-empai|em default true

add next to the frequency of IPI occurence per sample file the

emPAI value defined as [10**(#observed/#theoretical)] -1.

-md|matdesc default false

Add the description of the IPI id to the files generated

(matrices)

-all|allsample default false

Create for each peptide file a corresponding extended file

(.ext) containing the matched IPI ids and their description

**Authors**

Concept: Adalberto Costessi (Dept. Molecular Biology, NCMLS, Nijmegen, The Netherlands)

Implementation: Blaise Alako (Dept. Molecular Biology, NCMLS, Nijmegen, The Netherlands)

We want to thank Edwin Lasonder (CMBI-NCMLS, Nijmegen, The Netherlands) for suggesting to us the use of this analytical approach, which eventually led to developing this script
